# Oral Gastric Heterotopia: First Reported Case in the Hard Palate

**DOI:** 10.7759/cureus.52436

**Published:** 2024-01-17

**Authors:** Sofia Correia, João Mendes Abreu, Fátima Ramalhosa, Leonor Barroso, Isabel Amado

**Affiliations:** 1 Maxillofacial Surgery Department, Clinical and Academic Centre of Coimbra, Coimbra, PRT; 2 Stomatology Department, Clinical and Academic Centre of Coimbra, Coimbra, PRT; 3 Pathology Department, Clinical and Academic Centre of Coimbra, Coimbra, PRT

**Keywords:** oral pathology, palate, mouth, heterotopic tissue, choristoma, gastric heterotopia

## Abstract

Gastric heterotopia is characterized by the presence of mature gastric tissue outside the stomach, yet its occurrence in the palate has not been previously documented. We describe a case of gastric heterotopia in the hard palate of an elderly female patient, presenting as a swollen mass with associated secretion. Given the patient's age and clinical symptoms, a presumptive diagnosis of a malignant tumor originating from the minor salivary glands was made. An incisional biopsy of the mass revealed gastric heterotopia. Subsequently, the extended excision of the lesion was performed, leading to the full resolution of the patient’s symptoms. After a two-year follow-up period, no evidence of recurrence was observed. The importance of this case, underscored by the unprecedented location of gastric heterotopia, emphasizes the critical need for thorough evaluation to avert misdiagnosis, as well as the complete surgical excision of the lesion to prevent recurrence.

## Introduction

Gastric heterotopia (GH) describes the presence of mature gastric tissue located outside the stomach [1]. Occurring up to 2.6 times more frequently in men than in women, GH presents a higher prevalence during infancy or early childhood, although it may also be present in adults [2-4]. However, despite several proposed theories, the pathogenesis of these lesions remains unknown [5].

While GH may potentially affect any part of the gastrointestinal tract, oral presentation is exceptionally rare, primarily occurring in the tongue and the floor of the mouth [5,6]. Characterized by the absence of symptoms, GH tissue can occasionally swell, leading to highly variable symptoms depending on the affected location [2,6].

The differential diagnosis of GH is challenging and depends on factors such as the patient's age, the lesion's location, and its clinical characteristics. When occurring in the oral cavity, GH may resemble malignant or premalignant aggressive lesions, as well as benign entities [4,6]. Ultimately, the definitive diagnosis is determined by histological analysis, as this entity can exhibit similarities to the various parts of the gastrointestinal tract, including gastric, intestinal, or colonic mucosa [4]. Once diagnosed, despite GH usually having a benign course, surgical excision is recommended as the standard therapy. The incomplete removal of the mucosal lining, however, can result in recurrence [2,6,7].

Bains et al, in their report, included a comprehensive review of 68 documented cases of GH in the oral cavity, sourced from 57 reputable journals [6]. To the best of our knowledge, this review stands as the most exhaustive compilation available on this subject and, according to this and our literature search, we have found that GH has not yet been reported on the hard palate. This report details a previously unreported case of GH presenting as a mass in the hard palate of an elderly female patient.

## Case presentation

An 83-year-old woman presented with a mass in her left hard palate. The patient characterized the mass as painless and progressively growing over the preceding six months, accompanied by intermittent “bitter” drainage. The patient denied experiencing any systemic or constitutional symptoms and no additional signs or symptoms were described.

The patient’s medical history included gastroesophageal reflux disease (GERD), chronic gastritis positive for *Helicobacter pylori*, along with controlled hypertension and dyslipidemia, managed with angiotensin II receptor blocker (Telmisartan 40 mg) and hydroxymethylglutaryl-CoA (HMG-CoA) reductase inhibitor (pitavastatin 1 mg), respectively. No relevant surgical history was mentioned. No allergies, recent introduction of new medications, or history of drug or smoking habits were reported. Additionally, the family medical history was unremarkable. The patient also denied having undergone any dental treatment or oral trauma for the past six months.

**Figure 1:**
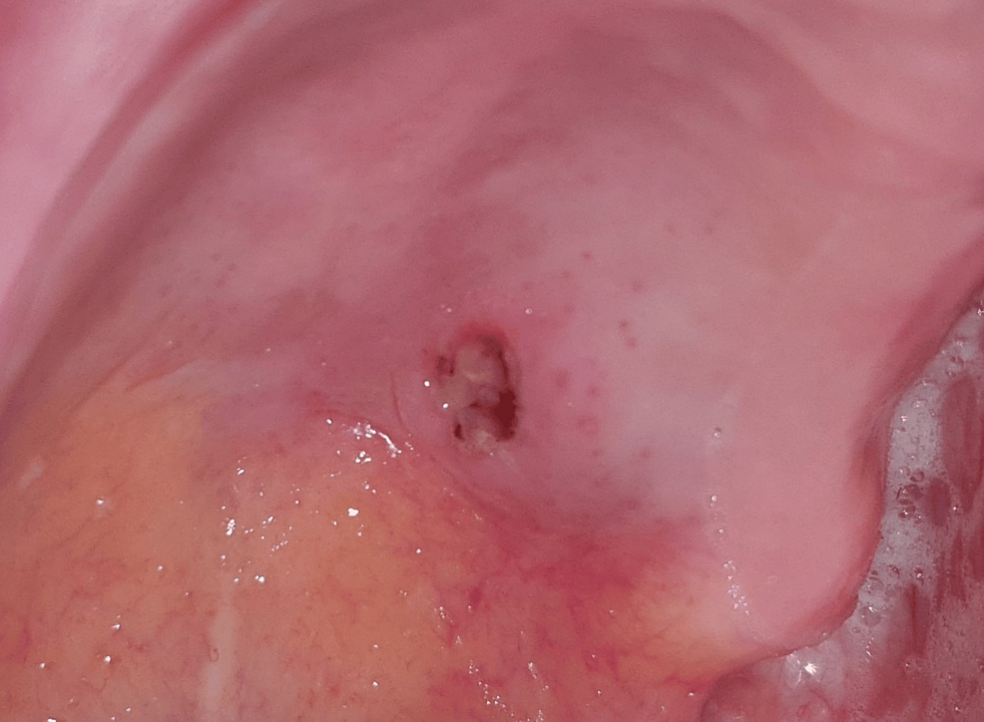
Palatal mass Visible in an intra-oral view, a nodular mass, approximately 2 cm in width, in the left hard palate of the 83-year-old patient, accompanied by a central ulcerative-like epithelial discontinuity.

Upon intra-oral clinical examination, a nodular mass was observed on the patient’s left hard palate, measuring 2 cm in width and positioned approximately 0.5 cm anterior to the hard and soft palate transition. The lesion also presented a central ulcerative-like epithelial discontinuity and drainage (Figure 1). Palpation of the lesion was deemed painless and demonstrated a fibroelastic consistency. Moreover, the patient revealed poor dental health, characterized by inadequate hygiene practices and multiple retained tooth roots in all four quadrants. Extra-orally, no facial or cervical swelling, masses, lumps, or other pertinent physical findings were observed or detected.

Considering the patient's clinical presentation, a differential diagnosis encompassing possibilities such as those of odontogenic origin and benign and malignant salivary gland neoplasms was proposed.

**Figure 2:**
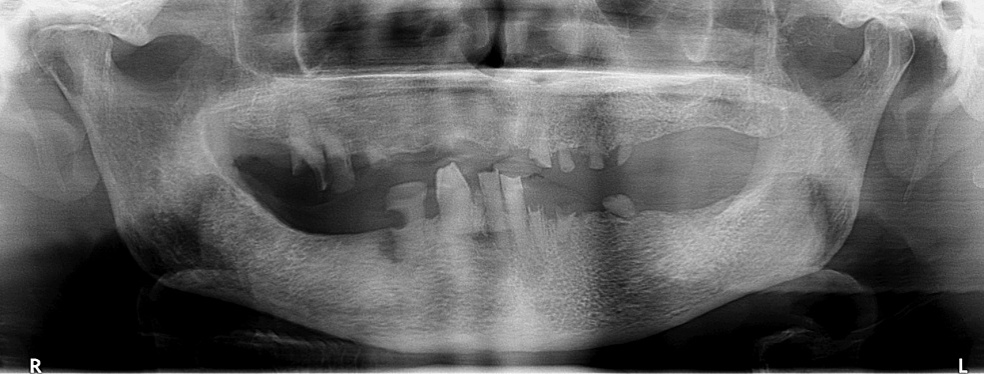
Panoramic X-ray showing multiple carious lesions and retained tooth roots

The patient was then informed about and consented to an orthopantomogram, ruling out an odontogenic origin (Figure 2). Subsequently, an incisional biopsy of the mass was performed revealing heterotopic (gastric) mucosa.

**Figure 3:**
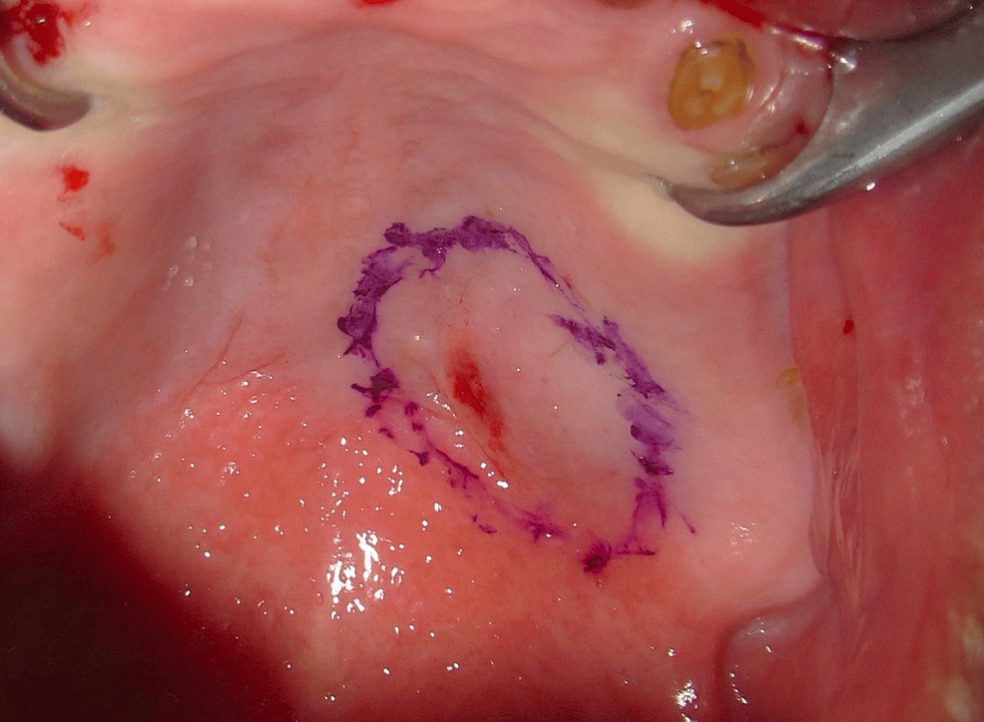
Intra-operatory intra-oral view of the palatal mass (pre-excision) with delineated surgical margins

As a result, a recommendation for the excision of the lesion to ensure complete clearance was proposed and accepted. The surgical margins were established by digitally outlining the fibroelastic mass (Figure 3).

**Figure 4:**
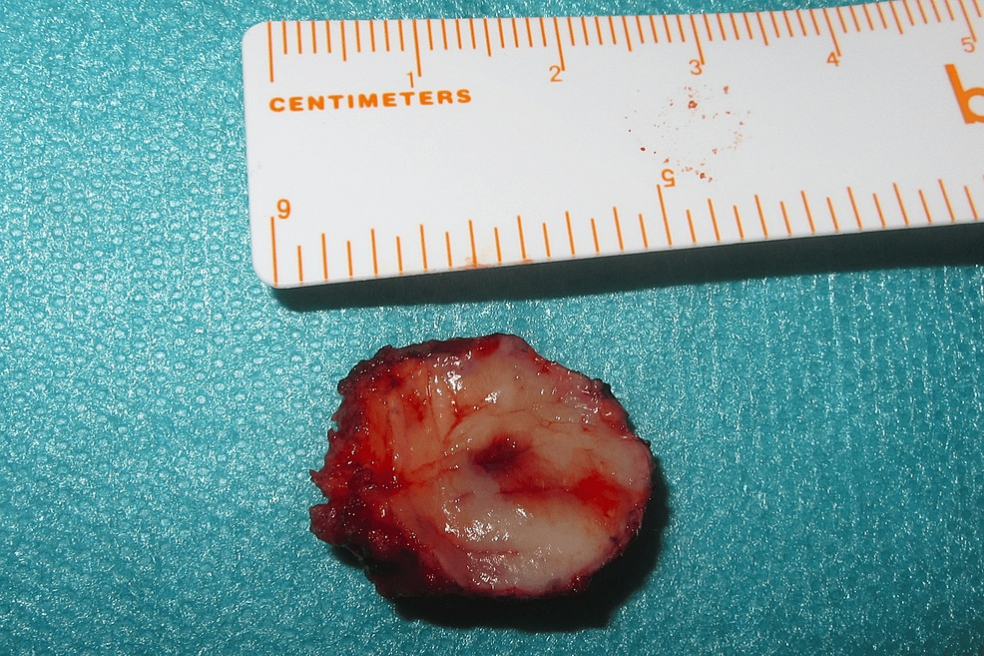
Post-excision view of the palatal mass

**Figure 5:**
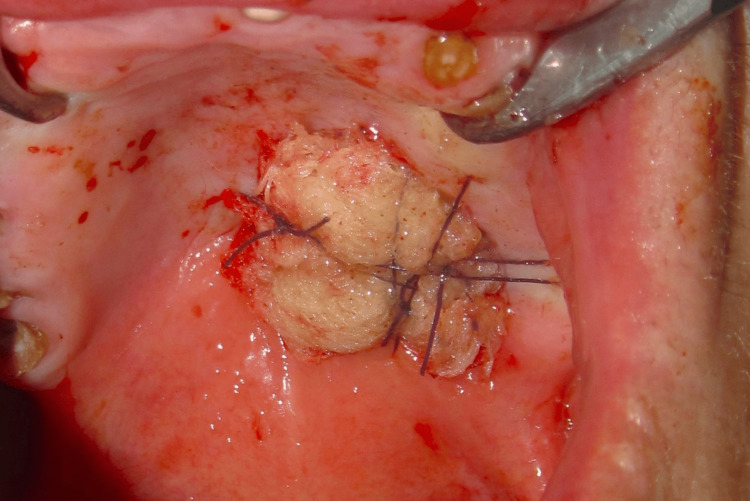
Intra-oral view showing a tie-over dressing with Vaseline-impregnated gauze covering the surgical site following the excision of the palatal mass

Throughout the procedure, the lesion exhibited cystic-like characteristics, notably segregated from the deeper palatal layers, and showed no extension into the bone. After the complete excision (Figure 4), closure was achieved by secondary intention with the application of a tie-over dressing with Vaseline-impregnated gauze (Figure 5). Additionally, all retained tooth roots were extracted.

**Figure 6:**
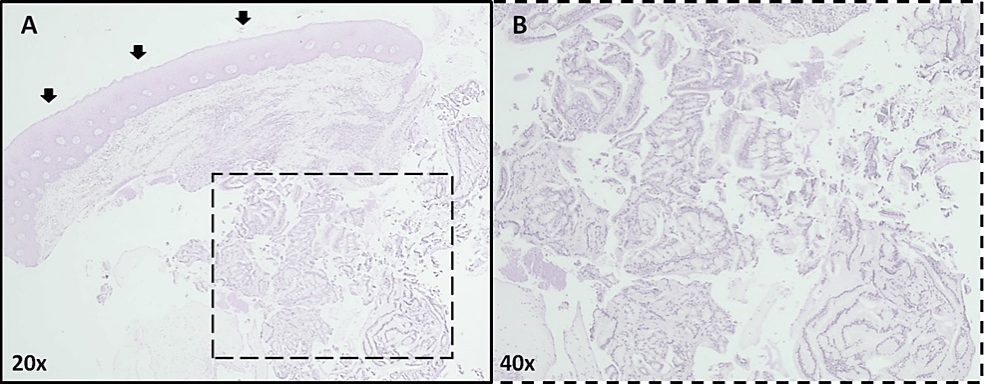
Gastric heterotopia of the hard palate (histological analysis with hematoxylin and eosin staining) A: At a 20x optical magnification, one can see a mucosal fragment from the hard palate, covered by a stratified, squamous, nonkeratinizing epithelium (arrow). Under the epithelium, a lesion is observed in the connective tissue (rectangle). B: At 40x optical magnification, one can see in detail the delineated rectangle mucinous epithelium, without dysplasia.

**Figure 7:**
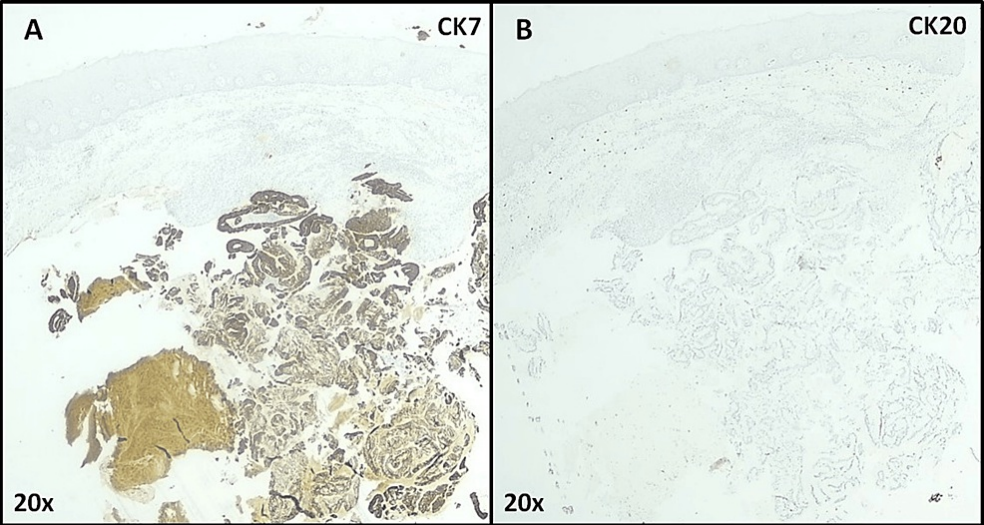
Gastric heterotopia of the hard palate (Immunohistochemistry with CK7 and Ck20 staining) At a 20x optical magnification, one can see the presence of secretory epithelium strongly positive for CK7 (A) and negative for CK20 (B). The CK 7 positive/CK 20 negative immunoexpression profile is common either in the salivary glands or gastric epithelium.

**Figure 8:**
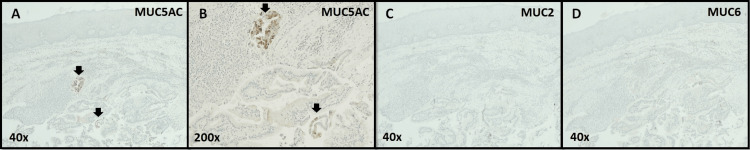
Gastric heterotopia of the hard palate (Immunohistochemistry with MUC5AC, MUC2 and MUC6 staining) A: At a 40x optical magnification, one can see the presence of focal positivity for MUC5AC (arrows), specific for gastric-like epithelium surface. B: At a 200x optical magnification, a more detailed view of the previous image is provided, characterized by the presence of columnar cells. C: In contrast, at 40x optical magnification, MUC2 immunohistochemistry shows an uncolored sample, indicating a lack of staining. D: Similarly, at 40x optical magnification, MUC6 immunohistochemistry also shows no impregnation.

A full histological examination of the mass then revealed a cystic lesion composed of gastrointestinal glands, without dysplasia (Figure 6), displaying strong positivity for CK7 (Figure 7) and focal positivity for MUC5AC (Figure 8), confirming, along with clinical evolution and imaging, the diagnosis as heterotopic gastric mucosa.

After surgery, a personalized maxillary soft plastic splint covering the entire palate was tailored for the patient to facilitate healing. However, the patient chose not to use it, citing adjustment issues and potential discomfort.

**Figure 9:**
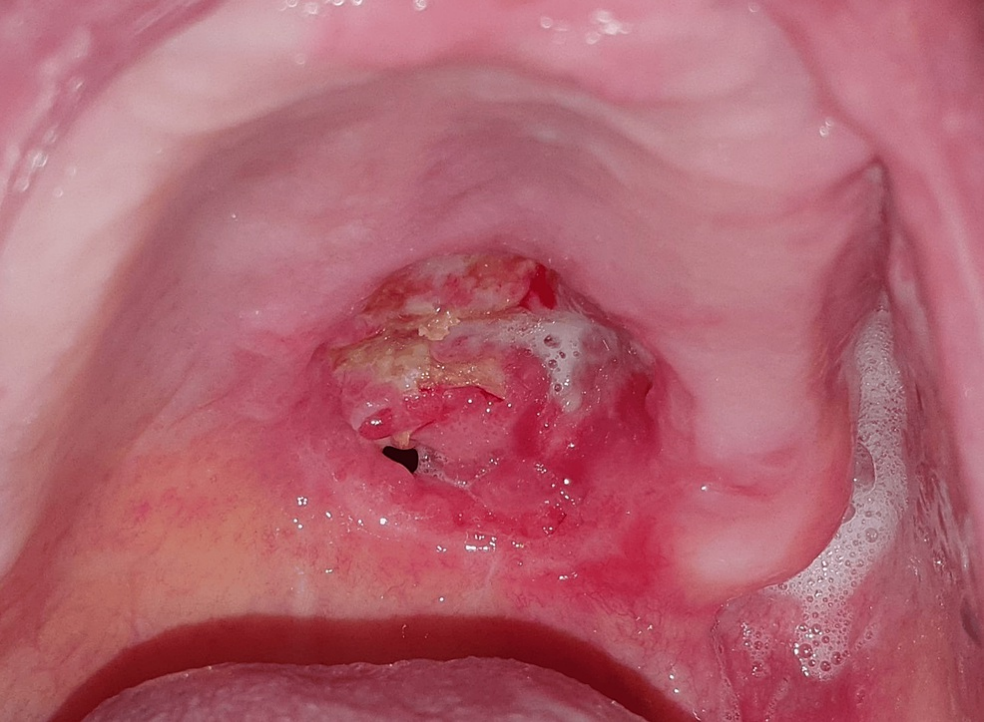
Post-excision status of the palatal gastric heterotopia mass (one-month follow-up) Visible in an intra-oral view, the surgical site shows abundant granulation tissue formation, compatible with a secondary healing process. Also visible is a 2 mm oral-nasal communication in the posterior surface of the wound.

Postoperatively, the patient exhibited favorable clinical progression, characterized by progressive and complete healing, as well as the resolution of initial complaints. However, during the one-month follow-up intra-oral examination, a 2 mm oral-nasal communication was detected at the posterior aspect of the wound (Figure 9). This resulted from an excision border tear that occurred when the patient resumed normal dietary habits. The patient reported minimal to no discomfort associated with the intake of food and liquids.

Considering the latter, a watchful waiting approach was adopted, reintroducing dietary restrictions (limiting intake to liquids and soft foods) and advising specific behavioral guidelines (such as avoiding exertion or Valsalva maneuvers) to aid the healing process.

**Figure 10:**
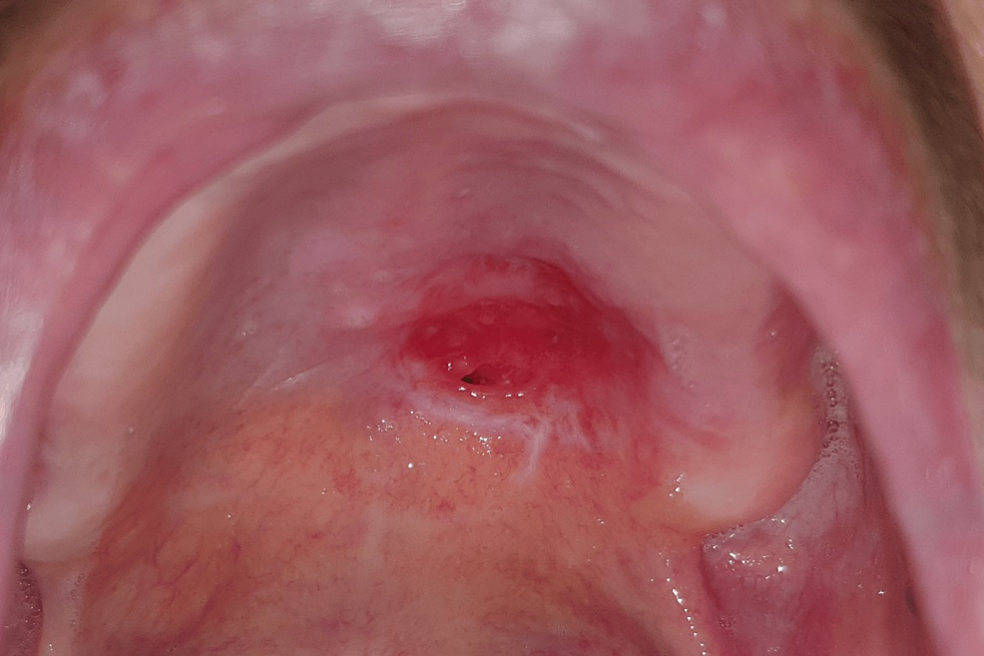
Post-excision status of the palatal gastric heterotopia mass (three-month follow-up) Intra-oral view shows a neo-mucosal tissue, occupying the totality of the surgical wound, accompanied by a successful and significant reduction of the oral-nasal communication.

**Figure 11:**
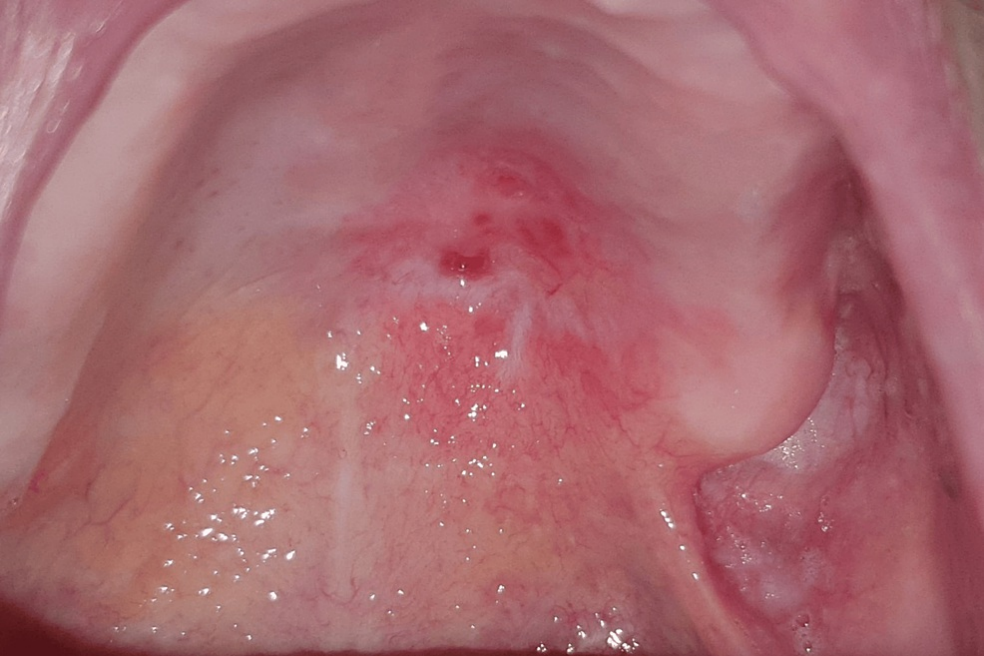
Post-excision status of the palatal gastric heterotopia mass (six-month follow-up) Intra-oral view shows a significant macroscopic normalization of the surgical wound, indicating successful healing, accompanied by a successful closure of the oral-nasal communication.

The subsequent follow-up consultations occurred at two months, three months (Figure 10), and six months (Figure 11) post surgery, revealing a progressive and spontaneous closure of the oral-nasal communication within the latter timeframe.

**Figure 12:**
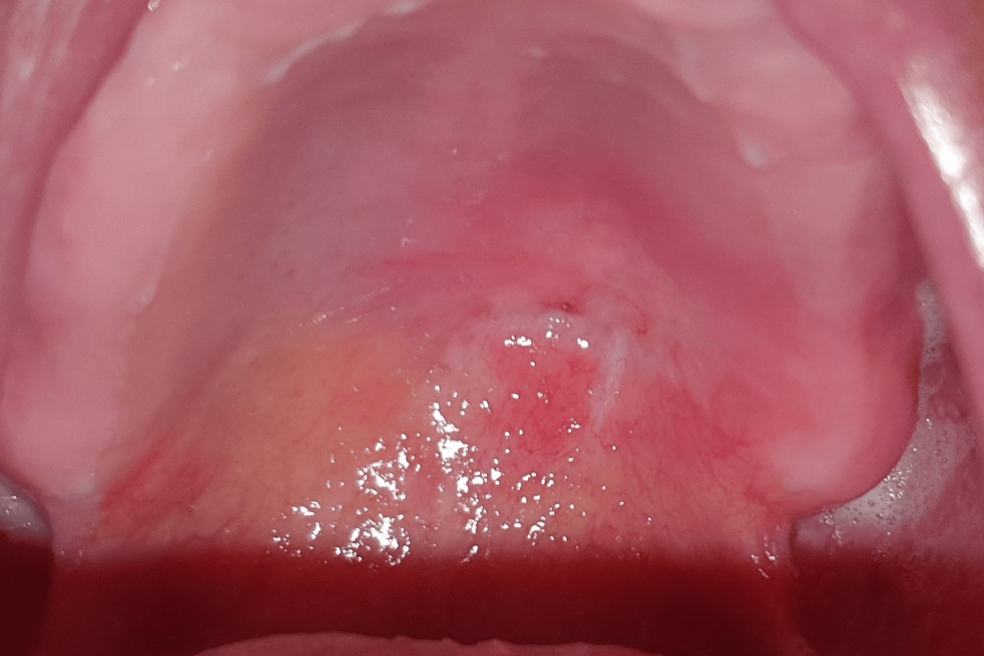
Post-excision status of the palatal gastric heterotopia mass (two-year follow-up) Visible in an intra-oral view, a macroscopic normal palate, with no clinical signs of recurrence of the gastric heterotopia mass.

The patient’s monitoring continued for two years until the discharge from the outpatient department was given, with no clinical signs or symptoms indicative of recurrence (Figure 12).

## Discussion

The existence of heterotopic gastric mucosa in the head and neck is an exceedingly uncommon phenomenon. Commonly termed choristomas, these lesions are defined as abnormal tissue masses that exhibit specific histological characteristics of a particular organ different from the one where they are situated [2].

Even though the cause of this condition remains unclear, some authors propose that its development might be linked to the presence of ectopic undifferentiated endodermal cells in the developing stomodeum. [4,6,8].

Although there is no conclusive evidence to support this, the authors postulate that there could be a connection between GH and the patient’s long-standing GERD. A possible mechanism is the migration and seeding of gastric cells in the oral mucosa, which would explain the presentation at an advanced age. If the seeding was of fundic cells, which are the acid-secreting cells of the gastric mucosa, this could be responsible for the complaint of “bitter” salivary secretion. However, this remains to be proven.

Gastric mucosal choristoma commonly presents as a cystic lesion, although solid forms have also been reported [2,9]. To date, cases of gastric mucosal choristoma affecting the hard palate have not been reported, with most occurrences being primarily located on the tongue and mouth floor, predominantly in children, and showing a slight male predilection [6,9-11].

We describe a unique case of a GH identified on the hard palate, highlighted by its occurrence in an elderly woman. In addition to its atypical nature, this report presents a symptomatic manifestation of GH, characterized by swelling and “bitter” drainage, which contrasts with the usual clinical presentation of these lesions, as they are typically asymptomatic and located more frequently in the thong or mouth floor [6].

Presenting itself as a 2 cm heterogeneous ill-defined ulcerative lesion, our primary objective was to exclude the possibility of a palatal tumor, given its frequent occurrence on the hard palate, prognosis, and macroscopically similar features [12].

Hard palate neoplasms are divided into malignant and benign forms, the latter including minor salivary gland tumors like pleomorphic adenoma, and mesenchymal tumors such as fibromas. Conversely, malignant varieties comprise squamous cell carcinomas, melanomas, lymphomas, sarcomas, and malignant tumors arising from minor salivary glands, such as adenocarcinoma, mucoepidermoid carcinoma, and adenoid cystic carcinoma. Nevertheless, despite their distinct pathophysiological processes, all the mentioned entities could exhibit various similarities to our patient's lesion [13].

Another diagnostic hypothesis, albeit less probable due to conflicting clinical characteristics, was a palatal mucocele. This condition encompasses both mucus retention cysts and mucus extravasation phenomena, differing from neoplastic growths, and stands as the most common disorder affecting minor salivary glands. However, contrary to hard palate neoplasms, mucoceles tend to have a very different clinical presentation from the depicted mass, growing asymptomatically with a bluish or pinkish smooth surface. These differences also extend to the absence of bitter drainage and the frequent relief of symptoms when ruptured [14].

For this purpose, an incisional biopsy was performed, ruling out the previous diagnosis and affirming the nature of the lesion as GH. A finding favored through the identification of a lining of stratified, squamous, non-keratinizing epithelium, with a cystic lesion composed of gastrointestinal glands, diagnostic criteria for GH [6,15]. Said conclusion was further supported by the immunohistochemistry findings, displaying a CK7+/CK20- along with focal MUC5AC+/MUC2-/MU6- profiles, duly framed by the clinical information and imaging.

After establishing the diagnosis, following the recommendations found in the available literature for GH lesions [2,6,7], surgical excision was proposed and accepted by the patient, with the full excision of the lesion successfully performed.

Following surgery, the patient showed a definitive resolution of symptoms, and the post-operative period proceeded without significant complications, with only a minor, almost asymptomatic, deferred oral-nasal communication reported, which was successfully addressed. Oral-nasal communications can be anticipated as potential complications following palatal surgery. Nevertheless, the management strategy is contingent on variables such as the size and precise location of the defect, the patient's age, and any concurrent comorbidities [16].

To mitigate the risk of recurrence linked to incomplete removal of the lesion, the patient underwent 24-month follow-up examinations before discharge. These assessments revealed no clinical evidence of recurrence, leading to a favorable prognosis.

## Conclusions

The documentation of, possibly, the first palatal GH case stands as an invaluable educational asset, providing profound insights for the medical community and significantly enriching our comprehension of the diverse spectrum of gastric heterotopia manifestations. This significance is highlighted by its macroscopic appearance, reminiscent of malignancy, thereby urging further evaluation and careful consideration to avoid misdiagnosis or unnecessary alarm.

Moreover, it underscores the paramount importance of achieving the complete surgical excision of the lesion, underlining the pivotal role of a comprehensive surgical intervention in ensuring successful management and minimizing the likelihood of recurrence or complications.

## References

[1] (2023). Small Intestine & Ampulla Congenital Anomalies: Heterotopic Gastric Mucosa. https://www.pathologyoutlines.com/topic/smallbowelheterotopicgastric.html.

[2] Chou LS, Hansen LS, Daniels TE (1991). Choristomas of the oral cavity: a review. Oral Surg Oral Med Oral Pathol.

[3] Erdem E, Tüz HH, Günhan O (2001). Gastric mucosal choristoma of the tongue and floor of the mouth. J Oral Maxillofac Surg.

[4] Martins F, Hiraki KR, Mimura MÂ, de Almeida Milani B, Gallottini M, Martins MT, de Sousa SO (2013). Heterotopic gastrointestinal mucosa in the oral cavity of adults. Oral Surg Oral Med Oral Pathol Oral Radiol.

[5] Drennen KC, Myers EN (1998). Heterotopic gastrointestinal mucosa of the oral cavity. Otolaryngol Head Neck Surg.

[6] Bains GK, Pilkington R, Stafford J, Bhatia S (2022). A case report of oral heterotopic gastrointestinal cysts (HGIC) and review of the literature. Oral Surg.

[7] Chai RL, Ozolek JA, Branstetter BF, Mehta DK, Simons JP (2011). Congenital choristomas of the oral cavity in children. Laryngoscope.

[8] Woolgar JA, Smith AJ (1988). Heterotopic gastrointestinal cyst of oral cavity: a developmental lesion?. Oral Surg Oral Med Oral Pathol.

[9] Martone CH, Wolf SM, Wesley RK (1992). Heterotopic gastrointestinal cyst of the oral cavity. J Oral Maxillofac Surg.

[10] Takato T, Itoh M, Yonehara Y (1990). Heterotopic gastrointestinal cyst of the oral cavity. Ann Plast Surg.

[11] Méndez Sáenz MA, de Jesús Villegas González M, Ponce Camacho MA, Cavazos Cavazos LM, Ibarra BS, Esquivel García BI, Treviño González JL (2016). Respiratory distress associated with heterotopic gastrointestinal cysts of the oral cavity: a case report. Ann Med Surg (Lond).

[12] Sarmento DJ, Morais ML, Costa AL, Silveira ÉJ (2016). Minor intraoral salivary gland tumors: a clinical-pathological study. Einstein (Sao Paulo).

[13] Aydil U, Kızıl Y, Bakkal FK, Köybaşıoğlu A, Uslu S (2014). Neoplasms of the hard palate. J Oral Maxillofac Surg.

[14] Abdel-Aziz M, Khalifa B, Nassar A, Kamel A, Naguib N, El-Tahan AR (2016). Mucocele of the hard palate in children. Int J Pediatr Otorhinolaryngol.

[15] Robinson L, Sengoatsi T, van Heerden WF (2021). Concomitant congenital intraoral dermoid cyst and heterotopic gastrointestinal cyst. Head Neck Pathol.

[16] Sahoo NK, Desai AP, Roy ID, Kulkarni V (2016). Oro-nasal communication. J Craniofac Surg.

